# Psychosocial Factors Associated with Musculoskeletal Disorders among Surgeons

**DOI:** 10.1192/j.eurpsy.2026.11065

**Published:** 2026-07-31

**Authors:** M. Bouhoula, M. Makhloufi, S. Ben Fradj, I. Misbah, I. Kacem, A. Chouchane, N. Gannoun, N. Belhadj, A. Aloui, H. Kalboussi, O. El Maalel, M. Maoua, S. Chatti

**Affiliations:** 1 Occupational Medicine department (LR19SP03), Farhat Hached University Hospital of Sousse, Tunisia; 2 Faculty of Medicine of Sousse, University of Sousse, Tunisia; 3 Epidemiology Department,, Farhat Hached University Hospital of Sousse, Tunisia; 4 Occupational Medicine department, Sahloul University Hospital of Sousse, Sousse, Tunisia

## Abstract

**Introduction:**

Surgeons are highly exposed to musculoskeletal disorders (MSDs), resulting from demanding physical tasks and significant psychosocial stressors.

**Objectives:**

To estimate the prevalence of MSDs among surgeons in Sousse (Tunisia) and to analyze their association with psychosocial risk factors.

**Methods:**

A cross-sectional study was conducted from July to December 2023 among surgeons at Sahloul and Farhat Hached University Hospitals. Data were collected using the Nordic Musculoskeletal Questionnaire and Karasek’s Job Content Questionnaire, including the social support dimension.

**Results:**

A total of 179 surgeons were included (mean age 35.7 years; 53.1% male). The 12-month prevalence of MSDs was 94.4%, with multi-site involvement in 48% of cases. The most affected regions were the lumbar spine (75.1%), cervical spine (62.7%), and shoulders (44.4%). The 7-day prevalence reached 97.2%, with a similar distribution. Psychosocial assessment showed that 86.6% of participants were classified as job strain, and 5.6% as iso-strain. Low decision latitude was significantly associated with cervical and lumbar MSDs (p=0.014; p=0.021). Low social support correlated with shoulder and dorsal spine disorders (p=0.011; p=0.026). Iso-strain was significantly linked to cervical spine and wrist involvement (p=0.047; p=0.006).

**Conclusions:**

MSDs are highly prevalent among surgeons and strongly associated with psychosocial stressors such as low autonomy, high demands, and poor social support. Preventive strategies should integrate ergonomic interventions with organizational changes that enhance decision latitude and promote team support.

**Disclosure of Interest:**

None Declared

